# Editorial for the Topic on Magnetic Materials and Devices

**DOI:** 10.3390/mi16070831

**Published:** 2025-07-21

**Authors:** Viktor Sverdlov

**Affiliations:** Christian Doppler Laboratory for Nonvolatile Magnetoresistive Memory and Logic, Institute for Microelectronics, TU Wien, Gußhausstraße 27-29, A-1040 Wien, Austria; sverdlov@iue.tuwien.ac.at

Magnets are materials that are characterized by their own persistent magnetic moments [1]. Nowadays, magnetic materials play an essential role in a broad range of modern technologies, from electronics to everyday devices to advanced sensors and medical equipment [2]. With the development of digital computing, magnets acquired a novel role as external data storage. The binary data is stored in the magnetic moment orientation, which can be altered by an external magnetic field [3]. The data can be extracted whenever needed by sensing the direction of the moment by using, i.e., the Giant Magnetoresistance Effect [4,5]. The magnetic moment and the data remain preserved for a very long time without external power. Because of several-year-long data retention, magnetic tape drives and hard disk drives were and are heavily used as main non-volatile storage by the data centers [6].

An electron, the main particle that defines the operation of electronic devices, also possesses the smallest intrinsic magnetic moment—the electron spin. Spintronics exploits both the charge and the spin of an electron. It benefits from the non-volatility offered by the magnetization and opens ways to create novel electronic devices with extended functionalities. A magnetic tunnel junction (MTJ) [7], a sandwich of two ferromagnetic layers separated by a tunnel barrier, is a representative example of such a device. Two states with parallel and anti-parallel ferromagnetic orientations are suited to store the binary data. The two states display different resistances. Thus, the information stored in an MTJ can be read by running a small current through it [8].

An MTJ is the main element of magnetoresistive random access memory (MRAM) [9]. At an earlier stage of the development, the information was written by means of an external magnetic field acting on one of the ferromagnetic layers in an MTJ—the free layer [10]. The real booster to MRAM development was the ability to write the information by purely electrical means without a magnetic field [11]. Spin-polarized electron currents running through the reference ferromagnetic layer of an MTJ deliver a spin–transfer torque (STT) [12,13]. The STT acts on the magnetization of the free layer and can flip it. The relative magnetization orientation of an MTJ is then altered by the electric current. This enabled the development of STT MRAM.

STT MRAM is fast, non-volatile, possesses a simple structure, has long retention, and has high endurance. MRAM is scalable beyond the flash memory limits [14], as major foundries are working towards implementing an 8 nm technology node and beyond [15]. To support MRAM development, reliable simulation tools are required. A finite element-based simulation environment capable of handling complex MTJ geometries composed of different magnetic and nonmagnetic materials is presented in [16]. Appropriate boundary conditions for the charge and spin current at the tunnel barrier interfaces enable the accurate reproduction of the torques acting on the free and reference layers in MTJs. The switching performance of modern MRAM cells composed of several ferromagnetic, tunneling, and nonmagnetic layers is addressed by solving the Landau–Lifshitz–Gilbert equation coupled to the spin and charge transport. The versatility of the finite element implementation is further demonstrated by investigating the switching acceleration in recently proposed advanced structures, including the double spin torque MTJs featuring two reference layers, as well as the ultra-scaled MRAM cells with elongated composite free layers [17,18].

STT MRAM is gaining momentum for embedded DRAM and low-level cache memory applications. However, STT MRAM is too slow to replace SRAM in high-level caches. To increase the speed of MRAM operation and to avoid large electron currents running through an MTJ, spin currents due to the spin Hall effect in heavy metals [19] are employed in spin–orbit torque (SOT) MRAM devices. Large spin currents impinging on the free layer from the heavy metal line generate a strong torque sufficient to manipulate the magnetization fast. At the same time, the strong electron current runs through the heavy metal and not through the MTJ. It does not damage the tunnel barrier of the MRAM cell. Recent advances in SOT MRAM are summarized in the review [20]. By including spin–orbit effects originating from the spin Hall and Rashba–Edelstein effects, the simulation environment [16] was extended [21] to model complex magnetization dynamics induced by spin–orbit torques in multi-layered SOT MRAM structures.

In magnetic racetrack memory [22], data is encoded in a series of magnetic domain walls (DWs) in a magnetic nanowire—the racetrack. Similar to a hard disk drive, the information is recorded by a magnetic head. DWs are moved along the magnetic racetrack by electric currents, enabling the data reading by a stationary read head. DWs in racetracks made of synthetic antiferromagnets move extremely fast [23]. Three-dimensional racetracks [24] may potentially enable memory concepts with high packing density and low energy consumption.

Non-volatile data retention, low leakage, and scalability, offered by spintronic devices, are extremely attractive for novel innovative logic architectures with the potential to outperform advanced complementary metal–oxide–semiconductor logic. A review of recent advances and remaining challenges of spin logic devices based on magnetic DW motion is presented in [25]. The data processing enabled by the DWs’ motion in the magnetic tracks can potentially simplify the complexity of logic circuits by performing several logic operations within the same device. Impressive advances in materials development for high-velocity DW motion driven by spin–orbit torques resulted in a successful demonstration of current-controlled DW logic gates. Combining DW logic gates with MTJs used to write and read the input and output data will enable the realization of compact and energy-efficient DW-driven spintronic logic circuits.

The development of magnetoresistive memories and spintronic logic devices requires the data transmission between them. The use of traditional interconnects carrying electric currents demands the data conversion from the magnetic to the electric domain and vice versa, which requires additional circuitry. To avoid this, interconnects should transmit magnetic signals. However, the magnetic signal rapidly decays while propagating. To suppress the damping, a novel type of interconnect with internal amplification is proposed [26]. Internal signal amplification is achieved by utilizing a composite multiferroic material. This way, the energy is transmitted from the electric to the magnetic domain, compensating for the attenuation of the magnetic signal. Numerical simulations indicate that the group velocity can reach 100 m/s with low energy dissipation, making the interconnects useful for a large variety of magnetic logic applications.

Leveraging the unique physical properties of piezoelectric and magnetostrictive materials at the mechanical resonance of bulk and surface acoustic modes, the review [27] demonstrates that the femto- to pico-Tesla sensitivity required for biomedical applications can be achieved. Exploring magnetoelectric materials for designing compact antennas at acoustic resonance is shown to be beneficial to reduce the antenna’s size while maintaining high radiation gain and efficiency. Future progress in designing magnetoelectric materials and devices with improved performance is predicted to expand their application towards quantum information technologies.

Efficient drug delivery to a particular target cell plays an increasingly important role in boosting the effectiveness of cancer therapies while minimizing the side effects on health. A novel microrobot specifically designed to perform a multiphase drug delivery is described in the article [28]. It is experimentally demonstrated that using the magnetic actuation of the magnetic liquid metal, the microrobot is capable of delivering drugs to target tissues. The drugs are then released via acoustic actuation, which destroys the bubbles with drugs embedded within a microtube. The proposed technology holds great promise for various applications in biomedical fields.

In summary, magnetic devices serve as key elements for various applications ranging from microelectronics and memory to sensors, quantum computing, and power devices. Magnetic devices are essential for advanced biomedical and health applications. They are indispensable for helping to enrich machinery functionalities, facilitate diagnostics, and enable targeted drug deliveries. Magnetic devices are inherent in improving the quality of everyday life.
